# Emerging Medications for Treatment-Resistant Depression: A Review with Perspective on Mechanisms and Challenges

**DOI:** 10.3390/brainsci15020161

**Published:** 2025-02-06

**Authors:** Michael J. Lucido, Boadie W. Dunlop

**Affiliations:** Department of Psychiatry and Behavioral Sciences, Emory University School of Medicine, Atlanta, GA 30322, USA; michael.john.lucido@emory.edu

**Keywords:** antidepressants, major depression, clinical trials, glutamate, biogenic monoamines, psychedelic, suicide, anhedonia, inflammation, TRD

## Abstract

**Background/Objectives**: Non-response to initial treatment options for major depressive disorder (MDD) is a common clinical challenge with profound deleterious impacts for affected patients. Few treatments have received regulatory approval for treatment-resistant depression (TRD). **Methods**: A systematic search of United States and European Union clinical trials registries was conducted to identify Phase II, III, or IV clinical trials, with a last update posted on or after 1 January 2020, that were evaluating medications for TRD. For both the US and EU registries, the condition term “treatment resistant depression” and associated lower-level terms (per registry search protocol) were used. For the US registry, a secondary search using the condition term “depressive disorders” and the modifying term “inadequate” was also performed to capture registrations not tagged as TRD. Two additional searches were also conducted in the US registry for the terms “suicide” and “anhedonia” as transdiagnostic targets of investigational medications. Trials were categorized based on the primary mechanism of action of the trial’s investigational medication. **Results**: Fifty clinical trials for TRD, 20 for anhedonia, and 25 for suicide were identified. Glutamate system modulation was the mechanism currently with the most compounds in development, including antagonists and allosteric modulators of NMDA receptors, AMPA receptors, metabotropic type 2/3 glutamate receptors, and intracellular effector molecules downstream of glutamate signaling. Psychedelics have seen the greatest surge among mechanistic targets in the past 5 years, however, with psilocybin in particular garnering significant attention. Other mechanisms included GABA modulators, monoamine modulators, anti-inflammatory/immune-modulating agents, and an orexin type 2 receptor antagonist. **Conclusions**: These investigations offer substantial promise for more efficacious and potentially personalized medication approaches for TRD. Challenges for detecting efficacy in TRD include the heterogeneity within the TRD population stemming from the presumed variety of biological dysfunctions underlying the disorder, comorbid disorders, chronic psychosocial stressors, and enduring effects of prior serotonergic antidepressant medication treatments.

## 1. Introduction

The emergence of selective serotonin reuptake inhibitors (SSRIs) and serotonin norepinephrine reuptake inhibitors (SNRIs) for major depressive disorder (MDD) in the late 1980s–90s offered greater safety, ease of use, and possibly efficacy for comorbid conditions compared to the older tricyclic (TCA) and monoamine oxidase inhibitor (MAOI) antidepressant medications (ADMs). With extensive industry marketing and the subsequent availability of generic formulations, SSRIs and SNRIs became ensconced, along with bupropion, as the “first-line” medications for MDD. In high-income countries, the previous 12-month use of ADMs by adults is currently estimated to be 4.7% [1]; in the USA, the past 30-day usage rate is 13.7%, which also includes those taking them for conditions other than MDD [2]. Over 80% of patients with MDD taking an ADM are receiving an SSRI or SNRI, and the proportion of patients receiving them as first-line treatments is likely even higher [3], consistent with numerous clinical practice guidelines that endorse this approach [4].

Monoamines such as serotonin modulate activity within neurocircuits and other signaling systems, but a primary dysfunction of monoamines is unlikely to underlie the etiology of most forms of MDD [5]. Nevertheless, monoaminergic ADMs are effective at reducing depressive symptoms, underscoring the important distinction between the etiology of MDD versus the mechanisms of action of its current treatments, which may be better conceptualized as biological “work arounds” to the core pathophysiology of the syndrome. Unfortunately, 40–50% of MDD patients fail to respond to serotonin-reuptake-inhibiting medications (SRIs) in clinical trials [6]. Although the concept of “treatment resistant depression” (TRD) has been discussed since the 1970s [7], the last decade has seen a substantial increase in drug development specifically targeting patients with TRD. Currently, the only medications that have US Food and Drug Administration (FDA) marketing indications specifically for TRD are esketamine (in conjunction with an oral ADM and, as of January 2025, as monotherapy) and an olanzapine–fluoxetine combination. Aripiprazole, brexpiprazole, quetiapine extended release (XR), and cariprazine have indications for use as adjunctive treatment for MDD [8,9,10,11], though it is only for aripiprazole and quetiapine XR that the indication specifies “with inadequate response to antidepressant therapy”. This paucity of approved treatments underscores a clear need to address the increased morbidity, health care utilization, and costs stemming from TRD [12].

Progress in developing new treatments has been hampered by the failure of the field to reach a standardized definition of TRD. There is an emerging consensus definition of TRD centered upon failure to achieve a response (≥50% symptomatic improvement from baseline) after two ADM trials at the minimum effective dose or higher during the current major depressive episode (MDE) [13,14]. The minimum duration of each medication trial has been proposed to be four weeks, based on the rapidly declining probability of subsequent response among non-responders at four weeks, though some have argued for 6–8 week trial minimums based in part on the finding that one-third of citalopram responders in the STAR-D trial first achieved response after week 6 [15]. Another issue of debate is whether the failed ADMs can be from the same class or must be from different classes, with evidence available to support both positions [16]. Notably, despite randomized controlled trials (RCTs) demonstrating similar efficacy of evidence-based psychotherapy and ADMs among outpatients with MDD [17], most current TRD definitions exclude the consideration of evidence-based psychotherapy failures [13,18], a notable deviation from the definition of treatment-resistant post-traumatic stress disorder (PTSD) [19] and the evidence that psychotherapy comprises a distinct mechanism of action for depression treatment [20].

**Figure 1:**
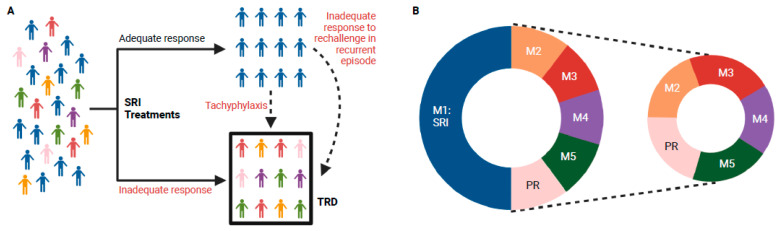
“Resistance” to drugs with a primary mechanism of serotonin reuptake inhibition (SRI) can result from three different treatment profiles: (1) failure to respond to initial serotonin reuptake inhibitor (SRI) medication trial, (2) loss of benefit during continuation or maintenance treatment (“tachyphylaxis”), or (3) failure to respond to re-initiated SRI treatment for a recurrent episode after prior successful SRI treatment (**A**). Part (**B**) of the figure is a schematic that uses M as an abbreviation for “mechanism of action”. M1 represents the MDD patients who are responsive to the SRI mechanism; M2–M5 stand for other subsets of the population that could respond to other (unnamed) mechanisms of action. After removal of SRI-responsive (M1) patients from an MDD population (approximately 50% of patients), the remaining TRD sample may be comprised of relatively small subsets of patients who can respond to specific drug mechanisms of action (M2–M5) and a proportion of pseudo-resistant (PR) patients, thereby increasing the difficulty of detecting statistically significant efficacy in clinical trials (**B**).

With this definition and current practice patterns, the overwhelming proportion of patients classified as having TRD are more precisely characterized as non-responsive to serotonin reuptake inhibition. However, even this subset of patients possesses complexities as SSRI/SNRI resistance can result from three different response profiles: (1) failure to respond to initial SRI treatment(s), (2) tachyphylaxis (“poop-out”) during continuation or maintenance treatment, and (3) failure to respond in a recurrent episode after a drug-free period subsequent to successful treatment with an SRI in a prior episode (Figure 1A). It is unknown whether the biological features of SRI resistance differ between the aforementioned response profiles or whether there are contributory iatrogenic effects resulting from long-term or repeated courses of ADM treatment [21,22,23]. It is remarkable that, given the widespread adoption of maintenance ADM treatment for MDD, so little research has explored these factors.

In terms of clinical care, the primary utility of designating a patient as having TRD is to inform the clinician to either (1) augment SRI treatments with a second treatment (medication or psychotherapy) or (2) switch from an SRI to an alternative mechanism of treatment, such as a medication with a different mechanism of action, psychotherapy, or a form of neuromodulatory/somatic treatment. Unfortunately, the categorical definition of TRD as two ADM failures does not provide information about which of the numerous next step options is likely to be helpful for a given individual patient, nor the array of mechanisms already tried and failed for a given patient. Several staging models of TRD have been proposed to address this issue [24], with only the Maudsley Staging Method establishing predictive utility [25]. To the extent that a TRD classification fails to inform clinical decision-making at the individual level, the TRD diagnosis suffers from similar limitations as the diagnosis of MDD: the broad heterogeneity (and presumed biological responsiveness) of individual patients is masked by group-level effects [23].

Conducting clinical trials targeting TRD faces many challenges [26]. Intuitively, it may seem that focusing on TRD would reduce the heterogeneity of a clinical trial sample to be evaluated; by excluding patients who are responsive to serotonin modulation, it may follow that a smaller number of pathological processes/biological response mechanisms remain, thereby increasing the probability of an alternative mechanism-of-action drug proving successful. However, because the response rate to SRI treatment for MDD is roughly 50–60% [17], sample heterogeneity may increase when moving from the global MDD population to the TRD population. Specifically, by removing the half of the MDD population with the capacity to respond to SRI treatment, TRD samples are enriched with patients whose depression may stem from an assortment of less common pathophysiologic processes, thereby resulting in a sample with even greater variability in treatment responsiveness (Figure 1B). Hence, clinical trials limited to a TRD population may face greater challenges in demonstrating efficacy than those targeting the MDD population as a whole, particularly if it is the case that SRI-responsive patients also possess the capacity to respond to non-SRI forms of intervention. 

Another concern for TRD trials is so-called “pseudo-resistance”, which refers to patients failing to improve with ADM treatment due to reasons other than biological non-response [27]. Drivers of pseudo-resistance include, though are not limited to, a psychiatric misdiagnosis of MDD (e.g., bipolar disorder or post-traumatic stress disorder), undiagnosed comorbidities (e.g., substance use disorder or neurocognitive disorder), overlooked medical comorbidities (e.g., obstructive sleep apnea), or simply poor medication adherence. Structured clinical interviews and assessments can help to alleviate some of these concerns, but it remains challenging to establish the adherence and quality of prior ADM treatment trials, which are crucial elements of the convergent definition of TRD described above. Secondary gain motivations of participants remain a significant concern in psychiatric clinical trials as well, as revealed by pharmacokinetic analyses [28] and survey evaluations of individuals enrolled in multiple trials [29]. Furthermore, although placebo response rates are lower in TRD studies than MDD, the response rates to active treatment are also typically lower [30]. Even among treatments showing promise in Phase 2, scaling into Phase 3 with multiple trial sites led by less-experienced or less-specialized investigators can introduce additional concerns for data validity [31]. Finally, novel TRD agents may be developed as monotherapy or augmentation treatments. Augmentation approaches may face the particular challenge of overcoming any deleterious effects of ongoing ADM (particularly SRI) treatment. For example, the efficacy of aripiprazole and brexpiprazole as augmenting (but not monotherapy) treatments may stem in part from their activity in overcoming the anti-dopaminergic effects of these agents in some patients [32]. Taken together, these factors present significant hurdles for establishing the efficacy of novel TRD-targeted medications.

An alternative approach to address treatment resistance or difficult-to-treat conditions is to focus on the transdiagnostic elements of disease, offering a way to cut through the heterogeneity that plagues our current classification system by targeting shared underlying processes in the development and maintenance of pathology [33]. The initial successes of this approach are most evident in the field of psychotherapy, where both universal (e.g., Unified Protocol for Transdiagnostic Treatment of Emotional Disorders) and modular (e.g., Modular Approach to Therapy for Anxiety, Depression, Trauma, or Conduct Problems [MATCH-ADTC]) interventions have demonstrated comparatively equivalent or superior efficacy in the treatment of varied mental health conditions to their disease-specific counterparts [34]. From a pharmacological perspective, the broad use of SRI medications may be viewed through a transdiagnostic lens; however, considerable effort continues to go toward a better understanding of the neurobiological substrate driving the phenotypic presentations observed in clinical practice, such as anhedonia or suicidal ideas and behavior. By harnessing these advancements, a more nuanced approach toward managing symptom clusters, such as changes to non-social interests, empathy, and cognitive focus [35], may be feasible. Despite this promise, though, there remains significantly more empirical support necessary to bolster this approach as a paradigm shift with real clinical utility [36].

This narrative review focuses on pharmacological agents currently in Phase 2, Phase 3, or Phase 4 trials for TRD, anhedonia, or suicidality. To contain the scope of the review, we do not report on other modalities of treatment for TRD, such as psychotherapies [37] or neuromodulation methods (including repetitive transcranial magnetic stimulation, electroconvulsive therapy, vagus nerve stimulation, and deep brain stimulation), which have been reviewed extensively elsewhere [38,39,40,41,42].

## 2. Materials and Methods

A systematic search of the US (https://clinicaltrials.gov/; accessed on 30 April 2022 and repeated on 15 December 2024) and EU clinical trials registries (https://www.clinicaltrialsregister.eu/, accessed on 30 April 2022 and repeated on 15 December 2024 ) was performed initially in April 2022 and repeated in December 2024. For both the US and EU registries, the condition term “treatment resistant depression” and associated lower-level terms (per registry search protocol) were used. For the US registry, a secondary search using the condition term “depressive disorders” and the modifying term “inadequate” was also performed to capture registrations not tagged as TRD. The search results from each were filtered to identify registrations with the last update posted on or after 1 January 2020. The results were saved and parsed to identify clinical trials utilizing a pharmacologic agent. Inclusion criteria included the following: trial inclusion criteria of unipolar TRD (as defined in the registered trial) or inadequate response to standard antidepressant treatment in MDD; Phase 2, 3, or 4 randomized controlled trial, open-label trial, or drug continuation trial using a pharmacologic agent as a monotherapy or add-on to standard ADM therapy; and trial was active after 1 January 2020. To be as inclusive as possible, we did not limit trial inclusion based on any specific definition of TRD or by requiring a minimum number of treatment failures; rather, we included any trial that indicated it was recruiting patients with unipolar TRD or MDD patients with an inadequate response to standard treatment. Exclusion criteria included the following: trial had been suspended, withdrawn, or terminated; trial had been completed; pharmacologic agent already FDA-approved or widely accepted for use in TRD; and non-pharmacologic trials (e.g., neuromodulation or psychotherapy without medication assistance). A subsequent search was conducted in December 2024 in the US registry using the terms “suicide” and “anhedonia” with the same filters to identify trials targeting these transdiagnostic elements. These initial results were filtered based on trial type (i.e., experimental/observational; phase) and status (i.e., suspended, terminated, or withdrawn), and the information for the remaining records was extracted manually and organized in a standardized format for screening and review.

## 3. Results

**Figure 2:**
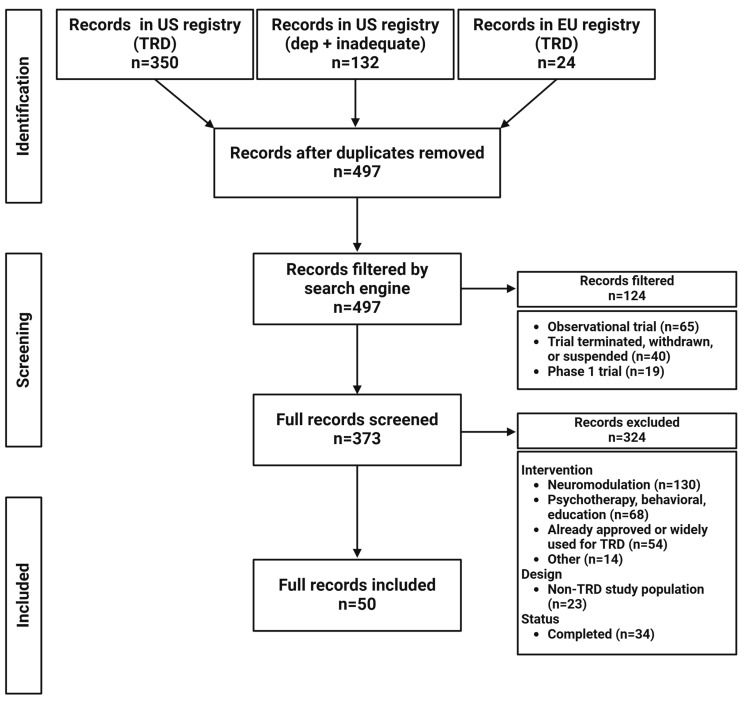
PRISMA flowchart for US and EU clinical trials registry results related to treatment-resistant depression (TRD). “Other” interventions marked for exclusion during the screening process included diet, dietary supplements without validated or listed components, behavioral complementary medicine (e.g., acupuncture), or in cases in which the intervention was a diagnostic test.

**Figure 3:**
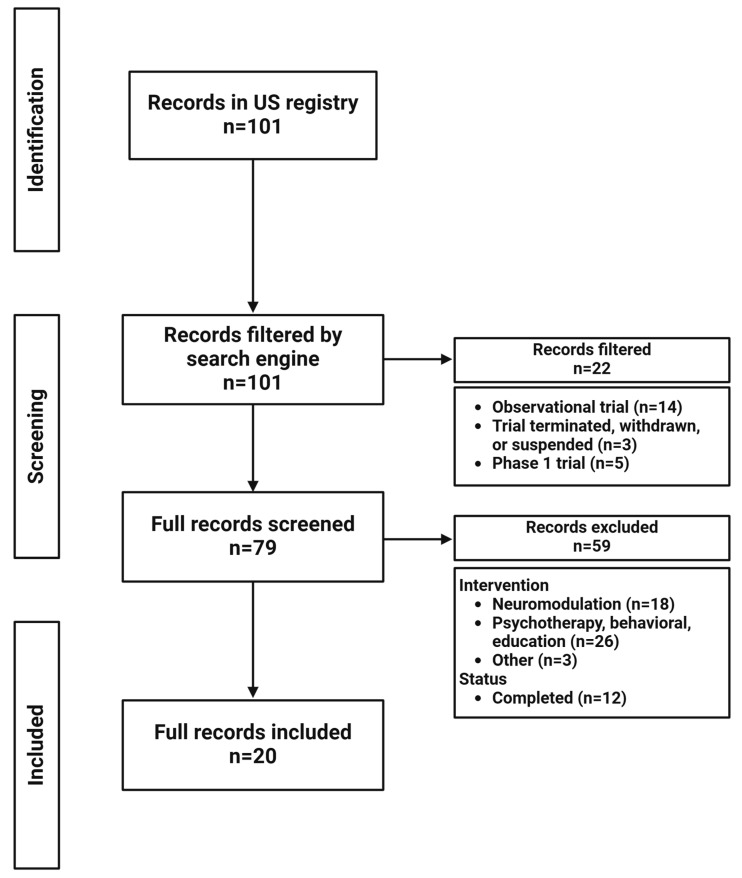
PRISMA flowchart for US clinical trials registry results related to anhedonia. “Other” interventions marked for exclusion during the screening process included diet, dietary supplements without validated or listed components, behavioral complementary medicine (e.g., acupuncture), or in cases in which the intervention was a diagnostic test.

**Figure 4:**
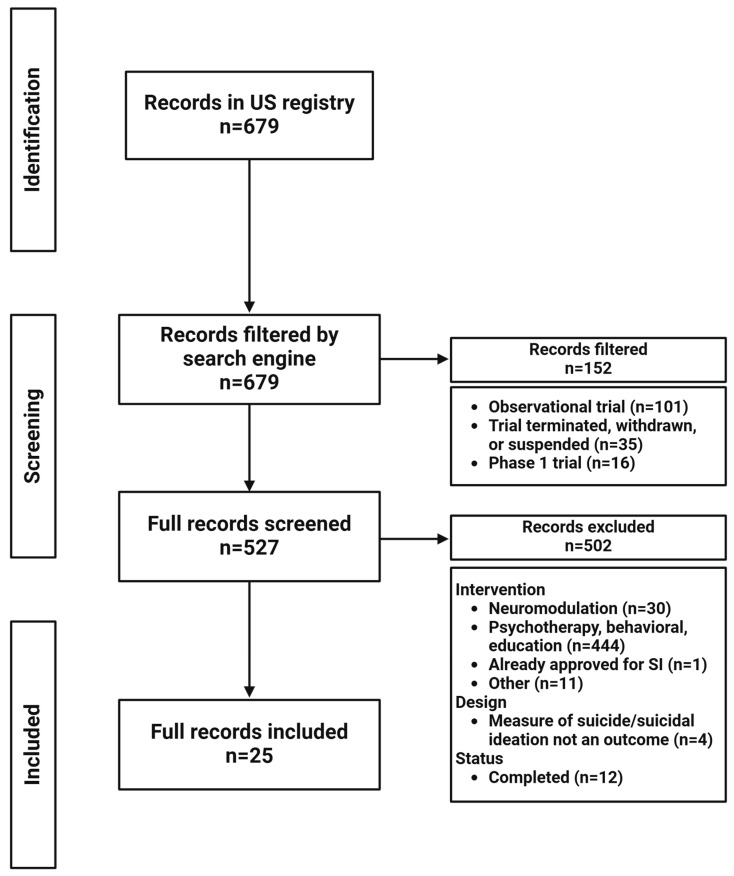
PRISMA flowchart for US clinical trials registry results related to suicide. “Other” interventions marked for exclusion during the screening process included diet, dietary supplements without validated or listed components, behavioral complementary medicine (e.g., acupuncture), or in cases in which the intervention was a diagnostic test.

Flowcharts for the results of the registry search terms are presented in Figure 2 (TRD), Figure 3 (anhedonia), and Figure 4 (suicide). Fifty clinical trials for TRD, 20 for anhedonia, and 25 for suicide were identified. The pharmacological mechanisms and characteristics of active clinical trials are summarized in Table 1, Table 2 and Table 3, grouped by pharmacological mechanism and agent for clarity. Given the scope of this review, trials marked as completed were also parsed to identify novel agents and provide a more nuanced view of what may emerge in the years ahead. 

Enhancing neuroplasticity has emerged as a common, convergent effect of depression interventions, including multiple classes of ADMs [43,44,45], psychotherapy [46], and electroconvulsive therapy [47,48]. The neurotrophic hypothesis of depression posits that reduced neurotrophic support results in neuronal atrophy, diminished neurogenesis in the hippocampus, and glial cell loss (particularly in glutamate-rich regions including the prefrontal cortex and hippocampus) [49,50,51,52], which together contribute to some, but not all, aspects of the depressed state [53]. ADMs oppose these effects [54] and in rodents their behavioral impact in many depression models is blocked when neurogenesis is prevented [55]. Neurogenesis has thus been adopted as a preclinical marker for antidepressant efficacy and as a potential target for novel ADMs [56]; however, it is notable that this is an area of debate with several lines of evidence suggesting that there is an absence or otherwise very low level of neurogenesis in the adult human [57,58,59]. Perhaps lending credence to the opposing view, drugs specifically developed through this pathway have yet to achieve marketing approval for MDD, and the rapid antidepressant effects of ketamine occur prior to the drug’s impact on neuroplasticity. However, while it is possible that the mood elevating effects of ketamine result from effects unrelated to neuroplastic change, such as via opioid signaling [60], the maintenance of mood improvement does appear to be mediated by the neuroplastic effects [45]. Although serotonergic, gamma-aminobutyric acid (GABA)-ergic, glucocorticoid, and inflammatory disease processes all impact neuroplasticity, to date, it is glutamate signaling and, more recently, psychedelic agents that have received the greatest focus for TRD drug development.

### 3.1. Glutamatergic Signaling Modulators

The successes of racemic (*R*,*S*)-ketamine and esketamine in treating TRD and acute suicidal ideation and behavior have invigorated a large investment into glutamate-targeted therapeutics for these clinical conditions [61,62]. As the primary excitatory neurotransmitter in humans, glutamate plays a vital role in neurodevelopment and a variety of neurobiological processes, including the mediation of cognition and emotion [63]. The abnormal regulation of glutamate and glutamatergic synapses in patients with MDD, particularly in corticolimbic neurocircuitry [64,65], supports a pathophysiological model of depression in which glutamatergic signaling is central. At the cellular/molecular level, reductions in glutamate transporter expression have been observed in preclinical models of depression [66] and clinical cohorts [67], implicating reduced glutamate turnover and subsequent increased extrasynaptic glutamate activity as a key component of the pathological state [68,69]. By enhancing glutamate-mediated increases in the release of neurotrophic factors, ketamine treatment increases synaptic density in key corticolimbic circuits [70].

Although extensive animal studies have revealed key components and time courses of ketamine’s antidepressant effects, a complete understanding of its antidepressant action remains to be elucidated. Ketamine’s best-established action is its uncompetitive N-methyl-D-aspartate receptor (NMDAR) antagonism. NMDARs are usually blocked by a magnesium or zinc ion in the receptor channel. With the neuronal depolarization and extracellular binding of its endogenous ligands (glutamate, aspartate, and glycine), the NMDAR channel opens and the magnesium or zinc ion leaves, thereby permitting sodium and calcium to enter the neuron. Thus, the activation of NMDARs is voltage (or use)-dependent. Ketamine blocks the receptor channel when it is open, causing a use-dependent inhibition of the receptor. The degree to which an antagonist remains in the receptor channel after it closes is referred to as “trapping”. High trapping NMDAR blockers, such as ketamine and phencyclidine, are those with slower release from the channel and are associated with greater cognitive and psychotomimetic side effects [71]. Partial trapping (or low affinity) antagonists include memantine, amantadine, dextromethorphan, and lanicemine (AZD6765). Theoretically, lower levels of trapping may enable a more selective blockade of neurons with high levels of tonic activity, such as GABAergic interneurons, preserving physiologic activity elsewhere [72] and reducing the cognitive and psychotomimetic effects of high trapping blockers like ketamine [73], which underlie its use as a drug of abuse.

Elucidating the mechanism(s) by which ketamine’s NMDAR blockade contributes to its antidepressant effect is crucial to advancing glutamate-targeted drug development. Several theories have been proposed, including the hypothesis that the selective antagonism of NMDARs on GABA-ergic interneurons by ketamine disinhibits glutamatergic neurons, thereby increasing glutamatergic firing and consequent α-amino-3-hydroxy-5-methyl-4-isoxazole propionic acid receptor (AMPAR) activation [74]. An alternative hypothesis posits that reduced NMDA-burst firing in the lateral habenula (which may be considered as an “anti-reward center”) disinhibits the release of monoamines from brainstem nuclei [75]. What does appear clear is that the sustained antidepressant action of ketamine depends upon BDNF, the expression of which is modulated via AMPA-receptor activation, as is the expression of the BDNF receptor (tropomyosin receptor kinase B, TrkB) and eukaryotic elongation factor 2 (eEF2), and the formation of the mechanistic target of rapamycin complex 1 (mTORC1)—all of which play roles in synaptogenesis and neural plasticity [76]. In the context of these vital roles the AMPAR plays in fostering the aforementioned processes, it appears that AMPAR activation may be a key mediator of the antidepressant efficacy observed with medications such as ketamine [77].

Despite their efficacy, the limitations of ketamine/esketamine (i.e., a typically short duration of benefit without repeat dosing, dissociation and cognitive impairments requiring transport home after treatment, and hypertensive and abuse risks) have spurred the search to develop molecules with improved risk/benefit profiles for TRD patients (Table 1).

AXS-05 (a combination drug comprised of dextromethorphan HBr 45 mg and bupropion HCl 105 mg extended release) received FDA approval for non-TRD MDD in August 2022. Dextromethorphan is an over-the-counter antitussive that functions as a low-affinity NMDAR antagonist with several other pharmacological targets, including sigma-1 receptor agonism and SERT inhibition [78]. However, its rapid metabolism by CYP2D6 during first-pass metabolism has limited its clinical utility. By co-administering a CYP2D6 inhibitor, such as quinidine in the dextromethorphan–quinidine combination drug marketed for the treatment of pseudobulbar affect, blood concentrations of dextromethorphan can be sustained for a clinically relevant duration [78]. Bupropion, itself an ADM, also inhibits CYP2D6, providing the rationale for the AXS-05 combination product. In a Phase 3 RCT evaluating 327 non-TRD MDD patients (the GEMINI trial), AXS-05 dosed twice daily for six weeks demonstrated superiority versus placebo [79], with statistical separation occurring at week 1. A subsequent trial in 80 non-TRD MDD patients compared AXS-05 to equivalent bupropion monotherapy (i.e., 105 mg BID) and also demonstrated the superiority of AXS-05, with sustained statistical separation beginning at week 2 [80]. However, in the STRIDE-1 trial, which evaluated AXS-05 versus bupropion monotherapy in 312 TRD patients, AXS-05 did not prove superior at the week 6 primary outcome time point, though it was statistically superior at week 1 and overall through the trial [81]. Another combination product, using deuterated dextromethorphan plus quinidine (AVP-786), failed to show significant antidepressant effects in controlled trials and has been repurposed for agitation management in dementia (NCT04408755, NCT02446132, and NCT03393520).

Another low-affinity, non-competitive NMDAR antagonist in Phase 3 testing for TRD is esmethadone (also known as dextromethadone, *d*-methadone, or REL-1017). Esmethadone has 20-fold lower potency at the mu opioid receptor than *l*-methadone (thus reducing abuse potential), has affinity for SERT and NET at micromolar concentrations, and has been demonstrated to increase circulating plasma BDNF in healthy subjects [82,83]. A Phase 2 trial of adjunctive oral esmethadone at 25 and 50 mg daily in 62 TRD patients demonstrated efficacy over placebo out to 7 days post-treatment [84], though the company developing this drug recently issued a press release indicating that an interim analysis of the ongoing Phase 3 trial indicated it is unlikely to meet its primary efficacy end point, raising uncertainty about its future development [85].

Nitrous oxide (N_2_0, “laughing gas”) is another non-competitive NDMAR antagonist, with additional inhibitory actions at AMPA, kainite, and nicotinic receptors. In the context of treating depression, nitrous oxide has typically been delivered in a 50% N_2_O/50% O_2_ mixture for one hour. In TRD samples, nitrous oxide has only been studied using a single dosing session, with a statistically significant benefit detectable two hours post dosing in an open label study [86] and two weeks post dosing for both 25% and 50% N_2_O concentrations versus a 0% N_2_O mixture in a 24-patient cross-over trial [87]. Achieving efficacy with lower N_2_O concentrations would improve the tolerability, which can be limited by nausea, vomiting, and headache. A third TRD trial with 44 patients, performed in China, is pending the release of results. Although nitrous oxide is a drug of abuse, typically in the form of “whippets” among youth, the drug is considered to have minimal addiction potential [88]. Due to its low solubility in blood, nitrous oxide clears rapidly, with most patients fully recovered in 10–15 min and able to drive home, a significant advantage over other dissociative agents. Xenon, another gas with NMDAR antagonist activity, is also being evaluated for efficacy in MDD patients with poor response to an ADM in an early Phase 1 trial (NCT03748446).

NMDARs are comprised of four subunits, the composition of which may have relevance as a target for TRD drug development. Ketamine has higher affinity relative to memantine for NMDARs containing the GluN2B/NR2B subunit [89], which is associated with basal levels of depression-like behavior in a mouse model [90]. Specific antagonists for NR2B-containing NMDAR, however, such as traxoprodil and rislenemdaz, have not proven successful in TRD clinical trials [91].

In contrast to orthosteric antagonists, which directly block the binding of a ligand to its receptor site, allosteric modulators of the NMDAR act at sites other than the agonist binding site, typically on the 7 trans-membrane domain, to decrease (negative allosteric modulator) or increase (positive allosteric modulator) activity induced by the endogenous ligand. Advantages of allosteric modulators versus orthosteric agonists or antagonists include improved selectivity for receptor subtypes, the reduced likelihood of receptor desensitization, and a broader ability to impact various aspects of receptor activity [92]. MIJ821 is a one such negative allosteric modulator of NR2B-containing NMDAR, which reduces the ability of glycine to bind to NMDAR. In a recent 6-week Phase 2 RCT that enrolled 70 TRD patients, adjunctive MIJ821 at 0.16 or 0.32 mg/kg delivered intravenously at weekly or biweekly intervals was found to be statistically superior to placebo at 24 and 48 h, though not at 6 weeks. The trial included an active control using ketamine 0.5 mg/kg weekly, and the MIJ821 arms had roughly similar effect sizes at all time points (NCT03756129) [93,94]. After acquisition by Novartis, the clinical trial assessing use in MDD patients with active suicidal intent was halted, leaving one Phase 2 trial underway in TRD. Whether negative allosteric modulators can avoid the psychotomimetic and dissociative effects of ketamine remains to be determined.

Positive allosteric modulators of NR2B-containing NMDAR, which unlike ketamine have the effect of increasing NMDAR signaling and consequently do not have associated psychotomimetic effects, have also been evaluated for efficacy in TRD with limited success. Among these is rapastinel (GLYX-13), which failed as an augmentation agent in three Phase 3 trials for patients with inadequate response to ADM [95] despite very encouraging Phase 2 data [96]. The development of its orally available derivative, apimostinel (NRX-1074), also appears to have ceased. Another positive allosteric modulator, AGN-241751, also recently failed to demonstrate efficacy in a large Phase 2 trial of non-TRD MDD patients [97]. Robust evidence has established that rapastinel promotes synaptic plasticity [98], indicating that such activity may be necessary but not sufficient for an antidepressant effect and underscoring the continuing uncertainties around how to modulate NMDAR activity for TRD. Another glycine-site antagonist, 4-chlorokynurenine (AV-101), recently failed to demonstrate efficacy in a 185-subject RCT evaluating its use as adjunctive treatment in TRD [99].

Another NMDAR allosteric modulator of interest is D-cycloserine (DCS). DCS is a glycine-site partial agonist that acts as a functional NMDAR antagonist at doses above 100 mg/day. Although numerous trials have indicated that DCS can enhance extinction learning for PTSD and anxiety disorders [100], data supporting its efficacy as an augmentation agent for TRD are weak [101]. One small placebo-controlled study found no benefit of 6 weeks of DCS (titrated to 1000 mg/day) for maintaining the antidepressant effect after response to ketamine treatment, though DCS did appear to provide protection against suicidal thoughts (see below). In the brain, DCS may function as a prodrug for D-serine, which is a full agonist at the NMDAR [102]. Cross-species studies have found that D-serine can improve sleep quality, cognitive flexibility, and memory retention [103]. D-serine has also shown mood-improving effects in healthy adults and can increase neurogenesis in mice, all of which suggest possible utility in TRD. Although no current studies are evaluating D-serine for TRD, these data suggest it may have utility for improving specific symptom components of TRD beyond that achieved with DCS [104]. Two factors that may limit D-serine as a therapeutic agent are its low bioavailability when given orally and its potential for nephrotoxicity at higher doses, which has been observed in rats [105].

Beyond the NMDAR, other druggable targets involved in glutamatergic signaling include ionotropic (AMPA and kainate) and metabotropic receptors (mGluR), as well as downstream effectors. Ketamine is not known to act through mGluR, but subtypes of this receptor family, particularly mGlu2/3 and mGlu5, have been implicated in mood regulation [106]. mGlu2/3 receptors are located primarily pre-synaptically, and their activation inhibits glutamate release. mGlu2/3 antagonists and negative allosteric modulators, such as decoglurant, have thus far failed to demonstrate efficacy in patients with MDD with an inadequate response to pharmacotherapy (EudraCT number 2011-002160-24) [107,108]. TS-101 is a mGluR2/3 orthosteric antagonist currently in Phase 2 trials. There do not appear to be any ongoing/active trials of mGluR5 modulators after several failures [109].

AMPARs differ from NMDARs in that their activation is not voltage-dependent, which enables them to regulate the voltage-dependent processes controlling calcium entry into neurons and activating second messenger systems involved in synaptogenesis [70]. Arketamine (PCN-101), the *R*-enantiomer of ketamine, appears to exert its effect primarily through AMPAR activation and, after demonstrating tolerability with limited dissociative effects in a Phase 1 study, moved to a Phase 2 clinical trial [110]. Unfortunately, while pilot data support its tolerability, its efficacy in treating the TRD population was not demonstrated [111], and a recent press release from the drug developer reported a failure to separate from the placebo in a three-arm Phase 2 trial of 102 TRD patients [112]. Preclinical data of a ketamine-metabolite, 2*R*,6*R*-hydroxynorketamine (HNK), found that it induces rapid and more sustained antidepressant effects than ketamine itself, likely through the indirect activation of AMPAR and potentially involving mGlu2 [113]. It is currently being investigated in a Phase 2 trial.

Finally, some investigational approaches are focusing on the downstream effectors of glutamatergic signaling, including the neurotrophin/TrkB signaling pathway and the integrative mTORC1 pathway. A leading candidate in this area is NV-5138, a synthetic leucine analog that selectively binds sestrin2 (a leucine amino acid sensor that activates the mTORC1 pathway) and skirts the rapid metabolism that leucine undergoes. It has demonstrated rapid antidepressant effects in a small clinical study [114] and is now in Phase 2.

### 3.2. Non-Glutamatergic Drugs

Although significant attention has been levied toward glutamatergic signaling as a target in TRD, the complexity of neurocircuits, neurotransmitters, and other signaling molecules in the context of depressive pathophysiology and treatment resistance offers a wide array of other potentially druggable targets (Table 2).

#### 3.2.1. GABAergic Modulators

A broader conceptualization of neurocircuit disruptions underlying depression incorporates an imbalance between excitatory (i.e., glutamatergic) and inhibitory (i.e., GABAergic) signaling [115]. GABA signaling mediates the stress response [116,117], a vulnerability factor for mood disorders [118,119], and preclinical and translational studies demonstrate depressive phenotypes in the context of dysfunctional GABA signaling [120]. GABA_A_ receptors are comprised of five subunits with differing subunit composition conveying different functionality. Depression has been associated with alterations in subunit composition yielding reduced GABAergic signaling [121]. Neurosteroids such as allopregnanolone, which are synthesized in the brain from progesterone, function as positive allosteric modulators of GABA_A_ receptors [122], though they also have a variety of other effects, including the negative allosteric modulation of nicotinic acetylcholine receptors and the 5-HT (5-hydroxytryptamine, commonly known as serotonin) 3 receptor, the inhibition of voltage-gated calcium channels, and the activation of pregnane X receptors [123,124,125,126]. Unlike the benzodiazepines, which engage synaptic γ-subunit-containing GABA_A_ receptors to increase the likelihood of phasic GABA-R activation [127], neurosteroids have a broader binding profile that includes not only synaptic GABA_A_ receptors but also the largely extrasynaptic, δ-subunit-containing GABA_A_ receptors that contribute to tonic GABA-mediated inhibition [128]. This difference in receptor subtype specificity and the consequent difference in mediating GABA inhibitory potentials appears to be central to the antidepressant effect observed in neuroactive steroids.

Brexanolone, an aqueous formulation of allopregnanolone, was approved by the FDA for the treatment of post-partum depression (PPD) in 2019 [122], though this formulation’s use is limited by requiring a monitored 60 h infusion. This was followed by the approval of zuranolone (SAGE-217), an orally bioavailable form of allopregnanolone, in 2023, for the treatment of PPD. Whether other forms of MDD not temporally associated with fluctuations in estradiol and progesterone (which may contribute to the development of PPD [122]) will also respond to neurosteroid treatments is not yet established. Although zuranolone, either as a monotherapy or when co-initiated with an ADM, demonstrated efficacy in non-TRD MDD patients [129,130,131,132], the FDA in 2023 declined to give marketing approval for zuranolone for the treatment of MDD, citing both efficacy and safety concerns [129]. Ganaxolone, a synthetic analog of allopregnanolone, received FDA approval in March 2022 for the treatment of seizures in patients with a rare genetic disorder. One small open-label study of ganaxolone as an adjunctive therapy in post-menopausal women with an inadequate response to treatment did demonstrate some efficacy over treatment as usual [133]; however, development has been limited as a function of null results in clinical trials focused on PPD and PTSD.

Another GABA-targeted agent is propofol, a general anesthetic that potentiates GABA and glycine receptors and inhibits NMDAR. An open-label study that administered EEG-guided personalized dosing of propofol on a thrice weekly regimen for a total of 10 infusions reported six of 10 TRD patients responded, with benefits lasting at least three months in most patients [134]. A Phase 2 RCT of 48 TRD patients testing high- versus low-dose propofol is currently underway (NCT03684447).

#### 3.2.2. Monoaminergic Modulators

The overall success of serotonin and norepinephrine modulation in the treatment of MDD led to concerted industry efforts to develop more targeted and specific pharmaceuticals in this vein. Most recently, the FDA in 2023 provided marketing approval for gepirone hydrochloride extended release, which acts as a 5-HT1a partial agonist, as a treatment for MDD [135]. Attempting to build off the efficacy of the norepinephrine and dopamine reuptake inhibitor (NDRI) bupropion as both a monotherapy and augmentation strategy, several companies pursued so-called triple reuptake inhibitors (TRIs), which inhibit the activity of the three monoamine transporters—serotonin (SERT), norepinephrine (NET), and dopamine (DAT) [136]. Despite massive investment, no TRIs have yet received marketing approval, and there have been several notable failures and/or abandoned candidate drugs (e.g., liafensine, tedatioxetine, and amitifadine). These failures provided insight into particular challenges for developing TRIs, such as identifying the optimal ratio of inhibition across the SERT, NET, and DAT that produces efficacy without abuse potential, quantifying transporter occupancy in vivo, the relative importance of phasic versus tonic signaling, and the complex interactions between serotonin, norepinephrine, and dopamine on their cell firing [136]. As such, industrial interest has waned in recent years, though two candidates still remain in advanced development. Liafensine, which had previously been investigated in MDD with null findings, has found renewed interest in the context of applied, proprietary pharmacogenomic biomarker use. The company developing this drug and proprietary pharmacogenomic biomarker recently released a press statement highlighting positive Phase 2 results and the approval of fast-track status from the FDA for further development [137]. Ansofaxine (also known as LY03005, toludesvenlafaxine, or 4-methylbenzoate desvenlafaxine) is rapidly metabolized in the periphery to desvenlafaxine (a marketed SNRI antidepressant) and demonstrates central nervous system penetration with hypothalamic concentrating and possibly DAT inhibitory activity [138]. Phase 2 and 3 clinical trials have demonstrated efficacy [139,140], and the drug gained regulatory approval for clinical use in non-TRD MDD in China in late 2022.

Lumateperone, an antipsychotic agent approved for the treatment of schizophrenia as well as depressive episodes associated with bipolar 1 and 2 disorders, has been bolstered by positive Phase 3 trial results as adjunctive therapy in MDD with inadequate response to antidepressant treatment [141]. Unlike its antipsychotic counterparts that already have regulatory approval as adjunctive treatments, lumateperone functions predominantly as a 5-HT2a antagonist with over 60-fold higher affinity compared to the D2 receptor. Evidence also points to its potential as a SERT inhibitor at higher doses, as well as an indirect, D1-mediated effect on NMDAR and AMPAR-mediated prefrontal cortical neurotransmission [142]. In terms of long-term safety and tolerability data, this receptor specificity profile seems to be translating to a notably improved side effect profile with little impact on cardiometabolic parameters, weight, prolactin levels, and motor disturbances equivalent to placebo and superior to comparator antipsychotics [142,143,144]. Though not entirely novel as an approach to TRD augmentation treatment, it may find use, if approved, over existing treatments based on the tolerability/safety profile. In a similar vein, OSU-6162 is a partial agonist at D2 and 5-HT2A receptors and has demonstrated anxiolytic properties and attenuated alcohol intake in rat models [145] and is in early development as a potential adjunctive treatment.

#### 3.2.3. Psychedelics

The classical tryptamine psychedelics (lysergic acid diethylamide, psilocybin, and dimethyltryptamine [DMT]) may also be considered as monoamine modulators based on their potent 5-HT2a agonism, though the degree to which their antidepressant effects are due to this direct biological effect rather than the array of psychological and mystical/spiritual effects remains an area of uncertainty [146]. Psychedelics share the property of producing intense experiences characterized by altered cognitive, emotional, and sensory processing as well as dramatic changes in brain connectivity [147] and the induction of neuroplasticity [148,149]. The leading current model of psychedelic-assisted therapy (PAT) involves providing several hours of preparatory psychotherapy sessions, one or more psychedelic dosing sessions, and then several hours of subsequent integration therapy sessions. The patient’s mindset (“set”) and the physical setting of the psychedelic experience are believed to be crucial for achieving therapeutic benefit [150].

The substantial efficacy of psychedelic therapy demonstrated in small trials of medically ill patients experiencing anxiety and depression in the face of end-of-life issues has encouraged their exploration as a treatment for MDD [151]. The potential advantages of psychedelic therapy approaches to TRD include good tolerability and safety, low abuse potential, potential efficacy for comorbid substance use disorders [152], the lack of need for chronic dosing, and the potential to impact important life domains beyond depressive symptoms. Psilocybin and its congeners are the psychedelic compounds most clinically advanced to date, with Phase 2 trials demonstrating efficacy in both TRD [153,154] and non-TRD MDD [155]. A Phase 2 RCT of 233 patients with TRD comparing 1, 10, and 25 mg of COMP360 (a proprietary, synthetic version of psilocybin) given in a single dosing session demonstrated efficacy for the 25 mg dose over the 1 mg dose at the week 3 primary outcome timepoint [153]. In a Phase 2 RCT of 104 non-TRD patients with MDD, a single dosing of 25 mg of psilocybin showed efficacy over a niacin placebo at the week 6 primary endpoint. Phase 3 programs are now underway for TRD using COMP360 and for MDD using psilocybin, with results expected in late 2025. Notably, in a 59-patient RCT comparing psilocybin to an active antidepressant (escitalopram), the psychedelic did not demonstrate superiority for depressive symptoms on the primary outcome, though numerous secondary measures indicated greater benefits for patients in the PAT arm [156]. Beyond psilocybin, several trials are evaluating the efficacy and safety of DMT (the psychedelic agent in ayahuasca) or 5-methoxy-DMT (5-MeO-DMT) for TRD (see Table 2). 5-MeO-DMT can be administered in a vaporized form, enabling more rapid time to onset and a shorter duration of altered mental status, properties which may increase the feasibility of its clinical application compared to psilocybin (which requires a minimum of 6 h post-dosing for the resolution of the altered mental state) [157].

A key challenge for psychedelic trials is the difficulty in identifying a placebo treatment that can maintain the blind after randomization due to the profound and rapid onset of psychedelic effects. The inadequate blinding of patients and investigators may heighten expectations for improvement after dosing. Niacin, which induces facial flushing, or a very low dose of the psychedelic has been utilized as a placebo, but a careful evaluation of unblinding and expectation effects has not been performed [158]. There are numerous preclinical research efforts underway exploring psychedelic analogs that may treat psychiatric symptoms without inducing the psychedelic experience, which would resolve the blinding issue and address the open question of whether the psychedelic experience itself is a necessary component for response to this class of drug [159,160].

Additional challenges for psychedelic treatments pertain to the uncertain durability of benefit, the need to identify patients with an increased risk of suffering harms from psychedelic exposure, and navigating the ethical and regulatory hurdles. Recent data suggest that some patients continue to demonstrate sustained improvements in mood for 6–12 months following psilocybin dosing [161,162], though the larger long-term follow-up samples from the Phase 3 trials will provide more reliable estimates of the durability of effect. Substantial efforts are being made to better quantify adverse effects from psychedelic treatment, including social and relationship harms that may not be identified adequately through standard adverse assessment methods [163,164]. There is also uncertainty around the psychotherapy to be co-administered with psychedelic treatments, including its theoretical orientation, delivery structure, and best ethical practices to ensure adequate patient consent and protection from exploitation [165,166]. Pharmaceutical regulatory bodies, such as the US FDA, have developed general guidance for psychotherapy delivery in conjunction with psychedelic dosing [167], but how these factors will be considered in determining the labeling for the regulatory approval of these drugs remains to be determined.

#### 3.2.4. Anti-Inflammatory and Immune Modulators

The immune system has long been hypothesized to play a role in the development or maintenance of psychiatric disorders, with the evolution of several prominent theories over the past 30 years [168]. There are three primary lines of evidence supporting this theory: (1) increased inflammation is present in patients with depression, (2) depressive symptoms may be induced with inflammatory stimuli, and (3) depressive symptoms may be reduced through the inhibition of inflammation. A recent meta-analysis estimated the prevalence of low-grade inflammation, defined as the elevation of the acute-phase reactant C-reactive protein (CRP) to >3 mg/L, to be nearly 30% in a sample of patients with MDD [169]. Patients with TRD appear to have more consistently elevated CRP levels when compared to healthy controls as well as treatment-responsive and untreated depressed patients [170]. Numerous prior studies have similarly demonstrated elevations in acute-phase reactants as well as other demonstrable markers of active inflammation in depressed patients, including the pro-inflammatory cytokines tumor necrosis factor (TNF), interleukin (IL)-6, and IL-17a [171,172]. Genomic and transcriptomic analyses of peripheral blood and cerebral spinal fluid have similarly shown increased inflammatory markers, including genes enriched for IL-6 and TNF signaling, as well as the evidence of enhanced immune trafficking and the activation of microglia [173]. There is an extensive literature on the induction of depressive symptoms by the administration of inflammatory cytokines such as interferon (IFN)-α [174,175] as well as vaccines and endotoxins [176,177,178] with significant phenotypic overlap with otherwise healthy depressed individuals [179].

Treatment with anti-inflammatory or immune-modulating agents has demonstrated potential to reduce depressive symptoms [180,181], and there is a recognized association between inflammatory status and diminished responsiveness to conventional first-line treatments in depression [182,183]. Both retrospective and prospective studies have demonstrated that elevated biomarkers of inflammation, including CRP, IL-6, and TNF, have been associated with a poor treatment response and repeated treatment failure [170,184,185,186,187], and transcriptomic studies have elucidated unique mRNA profiles in treatment-resistant vs. treatment-responsive patients [188]. Indeed, inflammatory status has been shown to predict responsiveness to electroconvulsive therapy and ketamine treatment [189,190,191], and, while the treatment of TRD patients with a TNF neutralizing antibody, infliximab, showed no overall differences between groups in terms of symptom improvement, post-hoc analysis demonstrated a significant interaction between the treatment and baseline level of inflammation (as measured by CRP) [192]. Employing inflammatory biomarkers as a potential predictor of treatment outcomes in MDD and TRD would provide a personalized and readily accessible approach to management, as exemplified in a recent small trial investigating the effect of the omega-3 fatty acid eicosapentaenoic acid (EPA) on depressive symptoms and inflammatory markers in an enriched patient pool [193].

The mechanism by which inflammation/immune dysregulation influences mood states is likely multifactorial and remains a matter of ongoing investigation. Some of the more prominent and robust evidence suggests changes to relevant neurotransmitter pathways [173]; an increased sympathetic tone via the activation of the hypothalamic–pituitary–adrenal (HPA) axis [194,195]; an over-activated inflammasome cascade [196,197]; an altered cellular and systemic metabolism and energy utilization [198,199,200]; and disrupted blood–brain barrier integrity [201]. Drug development efforts to overcome the effects of inflammation/immune dysregulation have primarily focused on canonical pro-inflammatory signaling cascades—IL-17 (ixekizumab), IL-6 (sirukumab, tofacitinib, baricitinib), TNF (golimumab, infliximab)—as well as both systemic mediators of inflammation (i.e., cyclooxygenase pathway) [183,202] and localized mediators of neuroinflammation (i.e., minocycline) [203]. While several medications remain under active investigation, either in TRD (ixekizumab, high-dose omega-3 fatty acids) or depressive symptoms in the context of systemic illness (e.g., baricitinib in rheumatoid arthritis, NCT05238896), many others have failed to consistently demonstrate efficacy (sirukumab, minocycline, and celecoxib) [173]. Negative results from these trials are unfortunately difficult to interpret for several reasons, including the following: (1) off-target effects for putative anti-inflammatory agents, (2) failure to prospectively enrich trials for increased baseline inflammation using accessible cut-offs (e.g., CRP level), and (3) failure to utilize transdiagnostic elements reliably demonstrated to be impacted by inflammation (e.g., anhedonic behavior and psychomotor speed and functional connectivity within reward and motor circuits) as outcome measures (discussed in detail in a recent review [173]).

An alternative strategy, based on accumulating evidence of the so-called “gut–brain axis” and its mediation through chemical messengers and the host inflammasome [204], seeks to harness the power of gut microbiota to treat neuropsychiatric disorders. Recent reports have identified specific commensals as having potential causal effects on the pathogenesis of depression [205] and that concentrations of gut-derived metabolites of tryptophan impact brain functional connectivity associated with anxiety [206]. Whether treatments targeting the gut microbiome, including fecal transplant, will prove enduring over time remains an important focus of this research.

#### 3.2.5. Opioid Receptor Modulators

The endogenous opioid system has been linked to various depression-related characteristics, including reward processing [207], attachment and social bonding [208], and the stress response [209]. Indeed, opioids (i.e., opium and morphine) have been in use for the management of “melancholia” and “mania” since antiquity and remained a popular therapeutic option well into the 20th century [210].

While the sparse sample of available studies using conventional µ-opioid receptor agonists and partial agonists (e.g., β-endorphin and buprenorphine) have demonstrated antidepressant effects [211,212,213,214], there exists a significant tension between efficacy and potential for abuse and dependence, as well as uncertainty related to durability and long-term outcomes, that has limited the potential for use in clinical practice. However, the µ-opioid receptor, which mediates the addictive potential of opioid agonists and their risk for respiratory depression, is only one component of the endogenous opioid system; there is growing pre-clinical and clinical evidence to suggest a rationale for targeting non-µ-opioid receptors, such as nociceptin and δ- and κ-opioid receptors [215]. An example of this approach is the combination of buprenorphine (partial µ-agonist and high-affinity δ- and κ-antagonist) plus a µ-antagonist, such as naltrexone or samidorphan, which effectively negates the µ-agonism and potentiates κ-antagonism, to augment standard ADM therapy [216,217]. Yet the early promise of these approaches has been tempered by null clinical trials, highlighted by the FDA declining to approve buprenorphine/samidorphan as an add-on treatment for TRD in 2019 [218]. The more direct κ-antagonist, aticaprant (CERC-501), also failed to meet statistical significance against placebo, though the trial had to be terminated early due to slow enrollment despite results trending positively (NCT01913535) [219]. Aticaprant does remain an active candidate, though the focus has shifted toward a more transdiagnostic view, targeting anhedonia/anhedonic depression. There are no current Phase 2 or 3 trials evaluating drugs for TRD primarily acting through opioid signaling, though an open-label Phase 4 trial of tianeptine, an atypical µ-agonist, is ongoing (NCT04249596).

#### 3.2.6. Additional Mechanisms

Seltorexant is a selective antagonist of orexin type 2 receptors, distinguishing it from the currently marketed orexin antagonists that non-selectively block both type 1 and 2 orexin receptors (e.g., suvorexant and lemborexant). Seltorexant has already demonstrated clinical efficacy in limited trials with TRD as an adjunctive treatment, with a more pronounced effect in patients who had a clinically relevant sleep disturbance at baseline [220,221]. The drug is now in Phase 3 trials for augmentation therapy in TRD (NCT04532749, NCT04513912, and NCT04533529). Notably, the preclinical data suggest that the antidepressant effects of orexin receptor antagonists do not depend on neurogenesis [222], which would be a departure from many of the current mainstream ideas about the physiology behind the efficacy of antidepressant medication.

Other potential treatments with active trials include S-adenosylmethionine, a co-substrate in enzymatic methyl group transfers; ABX-002, a thyroid hormone receptor agonist; and MAP4343, a microtubule-associated protein type-2 (MAP2) binding agent that enhances microtubule dynamics [223].

### 3.3. Transdiagnostic Approaches to Treatment and Application of Biomarkers

Achieving the goal of personalizing treatment for depression will require the identification of biomarkers that indicate whether a patient is more or less likely to respond to a given treatment, also known as prescriptive biomarkers [224]. These biomarkers may be more readily identified in TRD samples due to the filtering out of the high rate of placebo response in non-TRD MDD. There are currently no established peripheral biomarkers that can discriminate TRD as a diagnosis from non-treatment-resistant depression or that can provide an accurate prognosis of treatment outcomes [225,226,227]. Today, pharmacogenomic testing is the most evidence-based prescriptive biomarker for the selection of medication treatments for TRD, though the number needed to genotype to achieve one additional remission in TRD patients is approximately 19 [228,229].

There are two primary means for identifying prescriptive biomarkers. First, samples from randomized treatment trials can be retrospectively analyzed to identify a biomarker that significantly predicted differential outcomes to the intervention, which can then be prospectively tested in trials that stratify randomization to two or more treatments based on the biomarker. This approach has been used to evaluate EEG [230] and positron emission tomography biomarkers [231] and appears to have been successfully demonstrated in a Phase 2 trial with the TRI liafensine for which the sponsor has identified an undisclosed genetic biomarker, DGM4 (“Denovo Genomic Marker 4”), that consists of a single gene existing in a GG (DGM4-positive) vs. AA or AG (DGM4-negative) genotype (NCT05113771). This approach to personalization faces the challenge of patient sample heterogeneity between trials as variability in clinical or sociodemographic features may still significantly impact treatment response [231], though the process of utilizing sample enrichment and/or clearly defined states for biomarker application does appear promising.

A second approach is to identify patients with an abnormality in a biological system believed to underlie a component of mental dysfunction and then to prospectively test a treatment known to modulate that system in patients with that abnormality. These system abnormalities can exist across the current categorization of disorders (i.e., are transdiagnostic). Excessive fear processing [232], cognitive dysfunction [233], and anhedonia (both motivational and consummatory deficits) [234] are examples of transdiagnostic targets, with the latter emerging as the leading transdiagnostic target (Table 3), driven significantly by the neuroinflammation literature [173,235].

#### 3.3.1. Anhedonia

The neurobiological underpinnings of anhedonia involve dysfunctional networks including the corticostriatal reward circuitry/dopaminergic mesolimbic pathway and endogenous opioid signaling pathways [236,237,238]. Many drugs acting through these systems have been evaluated for anhedonia, including bupropion, carbidopa/levodopa, dextroamphetamine, and pramipexole, with mixed results in small samples [239]. More recently, several pro-dopaminergic drugs have been evaluated using the task-based neuroimaging of ventral striatal reactivity to reward anticipation in adults to demonstrate target validation for the treatment of anhedonia. Aticaprant (JNJ-67953964), a κ-opioid receptor antagonist that increases dopamine release in mesolimbic regions, produced significant enhancement on this imaging biomarker but unclear efficacy against anhedonia on clinical measures [240]. The other medication, ezogabine, is an opener of KCNQ2/3 potassium channels. In preclinical models, enhanced signaling through these channels in the ventral tegmental area protects mice against anhedonic behaviors [241]. In humans, ezogabine produced improvements specifically in anhedonic symptoms and an increase, not reaching statistical significance, in ventral striatal activation to reward anticipation [242]. While these studies are encouraging from a mechanistic perspective, the paradigm does not yet support the use of these specific imaging assessments as a prescriptive biomarker.

As an outgrowth of the larger collection of psychoneuroimmunology work focused on the impact of inflammation on the depressed state, there has been an interest in utilizing inflammation as a prescriptive biomarker to improve treatment selection in patients with an anhedonic MDD phenotype. One trial sought to harness the DAT inhibition of bupropion to promote dopamine neurotransmission within classic reward circuitry (ventral striatum to ventromedial prefrontal cortex) for patients with anhedonic depression and elevated CRP; however, there appeared to be no separation from the active comparator (escitalopram; NCT04352101). Using a similar paradigm, another study is assessing the impact of carbidopa/levodopa, the anti-Parkinsonian dopamine pro-drug, on resting state functional connectivity in classic reward circuitry and anhedonic depression using a CRP cut-off to enrich the patient cohort. Preliminary data are suggestive of a pattern of response in effort-based tasks and functional connectivity mediated by inflammation [243]. This study was recently expanded to determine if effects are appreciated in a larger patient population (NCT06075771). Baricitinib, a JAK1/2 inhibitor that dampens activation of the IL6 inflammatory cascade, is also being investigated for the treatment of anhedonic depression in HIV patients.

In a similar vein of enhancing dopamine neurotransmission in the context of systemic inflammation, several trials are being undertaken in Sweden to assess the efficacy of pramipexole, a D2/D3 agonist, as an adjunctive strategy in anhedonic depression. A pilot, open-label study did demonstrate efficacy and target engagement—improving depression and anhedonia scores as well as lowering mean CRP over 10 weeks of treatment [244]. A larger, double-blind, placebo-controlled trial is currently underway to follow up on these results (NCT05355337).

#### 3.3.2. Suicidality

Another important transdiagnostic target is suicidality [245,246,247,248,249], though the ethical and logistical difficulties inherent in studying such high-risk patients is a considerable barrier to the design of trials evaluating this transdiagnostic element [250]. The observed anti-suicidal effects of ketamine and esketamine have been a major boon to such efforts, however, and have encouraged the evaluation of several new or repurposed medications for this potential indication. As it relates to pathophysiology, suicidality has been associated with altered fronto-limbic connectivity involving the orbitofrontal cortex, anterior cingulate cortex, and thalamic regions [251,252,253]. While the treatments under investigation do not appear to necessarily specifically target these structures, they are associated with a more global disruption of connectivity. A possible exception to this may be opioidergic signaling, which can influence affective states through pain and social distress circuits that involve the thalamus and anterior cingulate cortex [254]. Indeed, the opioid partial agonist buprenorphine is among the repurposed medications being investigated, and the limited trial data available to date suggest that low-dose buprenorphine has efficacy in reducing depressive symptoms and suicidal ideation, though these trials did not examine changes in neural correlates, which could have supported the theorized pathophysiology [255].

Considering the prospective approach of the global disruption of connectivity, other NMDAR-modulating medications (nitrous oxide and MIJ821) and psychedelics (psilocybin) have been or are under evaluation. Among these, both nitrous oxide and psilocybin show promise for a relatively rapid change given the response observed in global depressive symptoms; however, MIJ821, an NR2B-containing NMDAR negative allosteric modulator, was apparently shelved in 2023 for this indication due to no new or changing safety signal in the interval analysis of the ongoing trial [256]. DCS, which has weak evidence as an augmenting agent in TRD and does not induce dissociative effects like the orthosteric NMDAR antagonists, is being tested as an augmenting strategy for transcranial magnetic stimulation (TMS) in suicidal youth/young adults (NCT06121284) and as a combination product (NRX-101) of high dose DCS (950 mg/day) with the atypical antipsychotic lurasidone for anti-suicidal effects in bipolar–depressed patients (NCT03395392).

## 4. Discussion

Success in identifying novel antidepressant mechanisms has been rare over the past three decades for a multitude of reasons, including but not limited to the tendency to model new drug candidates after the therapeutic actions of already approved drugs (a reverse translational approach), the use of pre-clinical models designed to be sensitive specifically to the actions of monoaminergic-based antidepressants as screeners for potential efficacy [70], and the challenges inherent in conducting clinical trials in TRD samples. An element of this latter issue that warrants further discussion is the variability in what defines a TRD sample. While the inclusion criteria for this review allowed for varied definitions of TRD (as defined by the trial investigators) to facilitate an expansive discussion of potential treatment strategies, it remains notable that, across the trials identified, there was inconsistency in how diagnostic eligibility was defined. For example, some trials explicitly include augmentation trials in the count toward the failed treatment tally (e.g., NCT06378229), some include a course of psychotherapy in the count (e.g., NCT04959253), some highlight that simple intolerance to a treatment may count toward the definition (e.g., NCT05383313), and some have upper limits on the number of failed trials (e.g., NCT05066672). Despite efforts to codify a TRD definition [14], this lack of consistency in active clinical trials does pose a problem for generalizability and future meta-analyses. As such, for a clinician examining the potential of a novel agent or a newly approved agent, clarity around the level of treatment resistance of the patients included in the trials of a specific medication will be needed to facilitate interpretation and translation of trial results to their own clinical practice. Additional concerns related to psychiatric clinical trial designs, which were not the focus of this review but have relevance for the real-world applications of clinical trials’ results, include the limitations of short follow-up periods, which may yield uncertainty about the durability of benefits [257,258]; small sample sizes, particularly in relation to genetics, neuroscience, and biomarker research [225,259,260]; and the limited diversity of participants, which can threaten the generalizability of trial results [261,262].

Despite such limitations, however, this review has identified many emerging medication approaches for the treatment of TRD, including several that may be nearing new drug application submission to the FDA as data come in from ongoing trials (i.e., seltorexant and psilocybin). Additionally, the orphan focus on addressing dimensional or transdiagnostic elements of patients’ presentations through pharmacology offers promise for clinical utility, exemplified by the regulatory approval for esketamine in the treatment of depressive symptoms in MDD accompanied by acute suicidal ideation or behavior. Considerably more evidence demonstrating linkages between phenotype, neural substrate, and biomarker will be necessary to support a larger clinical shift toward this approach.

When considering persisting clinical needs in the general treatment of MDD with current strategies, three that are readily apparent are reduction in time to clinical response, improvement in remission rates, and reduction in recurrence rates. Several of the medications already in use for TRD (i.e., ketamine and esketamine) do provide rapid symptomatic benefit, and others under investigation also seem to address this issue (e.g., nitrous oxide and psychedelics); however, what remains to be seen is the magnitude and durability of response. Improving remission rates may be best achieved by identifying biomarkers that predict outcomes to specific TRD treatments, given the variety of pathophysiologic mechanisms that may be present across the TRD population. Finally, the issues of the durability of benefit and reduction in recurrence are particularly salient for TRD given the high rate of depressive episode recurrence [263]. The assessment of recurrence as a matter of treatment success is at least in part muddied by the general paucity of data on the long-term, naturalistic course of MDD patients. The best data we have available on this matter, collected through the Collaborative Depression Study [264], are now more than two decades old, having followed patients receiving treatments that are uncommonly used in modern practice. While no investigational medications for TRD are currently seeking to address the durability element, future considerations may include a shifting focus to the development of medications that are not acutely acting antidepressants but rather medications that are most suitable for the maintenance phase of treatment. Possibilities in this area include buprenorphine or high-dose DCS after ketamine response [265], low-dose naltrexone for inflammatory-related depression [266], or corticotropin releasing factor receptor antagonists for stress-sensitive forms of depression [267].

Another unresolved question about TRD is the degree to which current treatment approaches for MDD iatrogenically contribute to its development [23]. Many patients who ultimately develop TRD evince good responses to ADMs earlier in their disease course, with a loss of benefit over time. Given the broad practice of open-ended maintenance ADM treatment to prevent new depressive episodes, it is remarkable how little research has explored the consequences of long-term treatment, particularly given the coincident and apparent rise in treatment resistance and tachyphylaxis. The belief that the loss of response (tachyphylaxis or “poop-out”) reflects underlying disease progression rather than a consequence of the sustained boosting of monoamine transmission remains an untested assumption that warrants careful examination. Although maintenance ADM treatment is clearly protective against depressive recurrence over 2 years of follow-up [268,269], whether this holds true over the longer term remains unexamined, as does the possibility that there exists a subset of adults with MDD vulnerable to developing treatment resistance due to long-term or repeated exposure to monoaminergic ADM treatments [21,22].

Many other agents currently in clinical trials for MDD may eventually demonstrate efficacy in TRD populations, even if their labeling does not indicate use for TRD specifically, much as MAOIs are used today. Clinicians will continue to face challenges in deciding between treatment options, for which the identification of clinical indicators or biomarkers for specific treatments would be enormously helpful. Post-marketing effectiveness studies to identify efficacious combinations of treatments are likely to become increasingly necessary as the number of approved treatments increases. Given the heterogeneity of TRD and the paucity of predictive preclinical models for its treatment, the successful development of new TRD treatments hinges heavily upon carefully designed, adequately powered, and rigorously conducted human trials.

## Figures and Tables

**Table 1:** Phase 2 or 3 clinical trials of glutamatergic signaling modulators for TRD or MDD with inadequate response to antidepressant medication that are ongoing or recently completed.

Drug/Compound	Proposed Mechanism	Clinical Trial Identifier(s)	Phase	Notes
Arketamine (PCN-101)	AMPAR agonist, sigma-1 agonist	NCT05414422	2	Adjunctive therapy;Failed to separate from placebo
AXS-05 (bupropion-dextromethorphan)	Uncompetitive NMDAR antagonist, sigma-1 agonist	NCT04634669, NCT04971291, NCT04039022, NCT02741791	3	Monotherapy; Approved as monotherapy for MDD in August 2022
Esketamine (CLE-100)	NMDAR antagonist	NCT04103892	2	Oral formulation; adjunctive therapy
Esmethadone (REL-1017)	NMDAR antagonist	NCT04855747, NCT03051256	3	RELIANCE studies; adjunctive therapy; unlikely to reach primary endpoint based on interim analysis Also evaluated as monotherapy or adjunctive for MDD (NCT05081167, NCT04855760)
(2R,6R)-hydroxynorketamine	Weak NMDAR antagonist, opioid receptor positive allosteric modulator	NCT06511908	2	Augmentation
MIJ821	NMDAR GluN2B negative allosteric modulator	NCT03756129, NCT05454410, NCT04722666	2	Adjunctive therapy
Nitrous oxide	NMDAR antagonist, iNOS inducer, cerebral vasodilator	NCT03283670, NCT05007028, NCT04957368	2	Monotherapy/adjunctive therapy; Also evaluated as monotherapy (NCT02139540, NCT03869736, NCT05357040)
NV-5138	Sestrin2 modulator, mTORC1 pathway activator	NCT05066672	2	Monotherapy
TS-161	mGluR2/3 antagonist	NCT04821271	2	Monotherapy

AMPAR, α-amino-3-hydroxy-5-methyl-4-isoxazole propionic acid receptor; iNOS, inducible nitric oxide synthase; MDD, major depressive disorder; mTORC1, mechanistic target of rapamycin complex 1; NMDAR, N-methyl-D-aspartate receptor; TRD, treatment-resistant depression.

**Table 2:** Phase 2 or 3 clinical trials of non-glutamatergic drugs for TRD or MDD with inadequate response to antidepressant medication that are ongoing or recently completed.

Drug/Compound	Proposed Mechanism	Clinical Trial Identifier(s)	Phase	Notes
GABAergic Modulators				
Propofol	GABA_A_ receptor positive allosteric modulator	NCT03684447	2/3	Not clearly defined as monotherapy vs. adjunctive therapy
Monoaminergic Modulators				
Ayahuasca	5-HT2 agonist, MAOI	NCT02914769	1/2	Response and remission rates significant at 7 days
DMT	5-HT2 agonist	NCT06524830	2	Monotherapy; administered as buccal film
5-MeO-DMT	5-HT2 agonist	NCT04698603, NCT05800860, NCT05660642, NCT05870540	2	Monotherapy; primarily open label studies
Liafensine	DAT, NET, SERT antagonist	NCT05113771	2	Monotherapy; FDA fast-track designation granted based on success of Phase 2 trial; currently in trial using a proprietary pharmacogenomic biomarker, DGM4
Lumateperone	5-HT2 antagonist, D2 antagonist	NCT05850689, NCT05061706, NCT04985942	3	Adjunctive therapy
OSU-6162	D2 and 5-HT2 partial agonist	NCT05641623	2	Adjunctive therapy
Psilocybin	5-HT2 agonist	NCT05220410, NCT05029466, NCT04519957, NCT06512220, NCT04670081, NCT05624268, NCT05711940, NCT06518720, NCT06132178, NCT06341426, NCT05383313, NCT05710237	2/3	Monotherapy or in conjunction with psychotherapy or neuromodulation; also evaluated as for monotherapy for non-TRD MDD (NCT03429075, NCT03866174)
Anti-inflammatory and Immune Modulators				
Eicosapentaenoic acid (EPA)	Anti-inflammatory omega-3 fatty acid	NCT05774665	2	Adjunctive therapy; 4 g of EPA-enriched omega-3 fatty acid supplement
Ixekizumab	IL-17A neutralizing antibody	NCT04979910	2	Adjunctive therapy; CRP > 2 mg/L as part of inclusion criteria
Tofacitinib	JAK1/3 inhibitor	NCT04141904	2	Adjunctive therapy; CRP > 1 mg/L as part of inclusion criteria. Trial terminated due to COVID with no new studies registered
Fecal microbiota transplant	Microbiome seeding	NCT04805879	3	Adjunctive therapy
Additional Mechanisms				
Seltorexant	Orexin type 2 receptor antagonist	NCT04951609, NCT04532749, NCT04513912, NCT04533529, NCT06559306	3	Adjunctive therapy

5-HT2, serotonin receptor type 2; GABA_A_, gamma-aminobutyric acid type A receptor; IL-17A, interleukin 17A; JAK1/3, Janus Kinase type 1 and 3; MDD, major depressive disorder; TRD, treatment-resistant depression.

**Table 3:** Active or recently completed clinical trials targeting anhedonia or suicidal ideation/suicidality.

Drug/Compound	Proposed Mechanism	Clinical Trial Identifier(s)	Phase	Notes
Transdiagnostic Approach—Anhedonia				
Aticaprant	Κ-opioid receptor antagonist	NCT05455684, NCT05455684, NCT05550532, NCT06514742	3	MDD + moderate to severe anhedonia with inadequate response to SSRI or SNRI
ALTO-203	H3 inverse agonist	NCT06391593	2	MDD + anhedonia; monotherapy
Ansofaxine	DAT, NET, SERT antagonist	NCT06270433	NR	MDD + anhedonia; using desvenlafaxine as an active comparator
Baricitinib	JAK1/2 inhibitor	NCT05849038	2	HIV with MDD + anhedonia
Buspirone	5-HT1A agonist	NCT05357547	2	Reward processing in healthy controls; trial completed in 2023 with no results posted to date
Estradiol	Estrogen receptor agonist	NCT05282277, NCT06610305	4	First study investigating anhedonia and/or psychosis in perimenopausal women; second study investigating effect of estradiol vs. progesterone vs. placebo on MDD with perimenstrual symptom worsening + anhedonia
Insulin	Glucose regulation	NCT03915613	1/2	Proof of concept study establishing a role for insulin/insulin resistance in anhedonia and behavioral deficits in MDD
NBI-1065846 (TAK-041)	GPR139 agonist	NCT05165394	2	MDD + anhedonia; no separation from placebo on measures of anhedonia or overall depression. Also evaluated in schizophrenia + anhedonia (negative results; NCT03319953); no further trials listed
Pitolisant	H3 agonist, sigma1 agonist	NCT05849675	NR	Healthy volunteers; assessing effect on functional connectivity.
Pramipexole	D3 receptor agonist	NCT05355337, NCT05825235	4	MDD + anhedonia
Psilocybin	5-HT2 agonist	NCT06230757	2	TRD + anhedonia
Sinemet (carbidopa/levodopa)	Dopamine agonist	NCT06075771, NCT03243552, NCT05909267	2/4	MDD + anhedonia; CRP > 2 mg/L as inclusion criteria; second study investigating ASD with social anhedonia and the impact of levodopa vs. placebo and social skills training third study investigating impact in MDD + anhedonia, no CRP cutoff listed
XEN1101	KCNQ2/3 agonist	NCT04827901	2	MDD + anhedonia completed in 2024 with no results posted to date
Transdiagnostic Approach—Suicidal Ideation				
Buprenorphine	Partial mu agonist	NCT04116528, NCT03646058	3	First study investigating ketamine followed by buprenorphine for sustained anti-suicidal effect; second study investigating analgesic buprenorphine dosing as augmentation to antidepressant treatment in acute suicidality
Estradiol	Estrogen receptor agonist	NCT04112368, NCT03498313, NCT06191289	2/4	Treatment of peri-menstrual suicidal ideation
D-cycloserine	NMDAR glycine site partial agonist	NCT06121284	2	Augmentation of intermittent theta burst rTMS
Ketamine	NMDAR antagonist	NCT04669665, NCT06366334, NCT05468840	2/3/4	MDD + current suicidal ideation; active suicidal ideation in the emergency room setting; suicidality in opiate-use disorder; Ten additional active studies
Nitrous oxide	NMDAR antagonist, iNOS inducer, cerebral vasodilator	NCT05710887, NCT06430489, NCT06636357	2	Non-psychotic MDD + acute suicidality in ED setting
NRX-101 (D-cycloserine + lurasidone)	NMDAR glycine site partial agonist + antipsychotic	NCT03395392, NCT03396068	2	Bipolar depression + sub-acute suicidal ideation/behavior
Psilocybin	5-HT2 agonist	NCT05220410	2	TRD + chronic suicidal ideation; open label
Tetrahydrocannabinol	CB receptor agonist	NCT06381180	1/2	PTSD with chronic risk for suicidality

5-HT1A, serotonin receptor type 1A; CB, cannabinoid; CRP, C-reactive protein; D3: dopamine type 3 receptor; GPR139, G-protein coupled receptor 139; MDD, major depressive disorder; rTMS, repetitive transcranial magnetic stimulation; TRD, treatment-resistant depression; NR, not reported.

## Data Availability

The original contributions presented in this study are included in the article. Further inquiries can be directed to the corresponding author.

## Abbreviations

The following abbreviations are used in this manuscript:

SSRI	selective serotonin reuptake inhibitor
SNRI	serotonin norepinephrine reuptake inhibitor
MDD	major depressive disorder
TCA	tricyclic antidepressant
MAOI	monoamine oxidase inhibitor
ADM	antidepressant medication
SRI	serotonin reuptake inhibitor
TRD	treatment-resistant depression
FDA	Food and Drug Administration
XR	extended release
MDE	major depressive episode
RCT	randomized controlled trial
PTSD	post-traumatic stress disorder
GABA	gamma-aminobutyric acid
NMDAR	N-methyl-D-aspartate receptor
AMPAR	α-amino-3-hydroxy-5-methyl-4-isoxazole propionic acid receptor
TrkB	tropomyosin receptor kinase B
eEF2	eukaryotic elongation factor 2
mTORC1	mammalian target of rapamycin complex 1
DCS	d-cycloserine
mGluR	metabotropic glutamate receptor
HNK	2R,6R-hydroxynorketamine
5-HT	5-hydroxytryptamine, serotonin
PPD	post-partum depression
NDRI	norepinephrine and dopamine reuptake inhibitor
SERT	serotonin transporter
NET	norepinephrine transporter
DAT	dopamine transporter
DMT	dimethyltryptamine
PAT	psychedelic-assisted therapy
5-MeO-DMT	5-methoxy-dimethyltryptamine
CRP	C-reactive protein
TNF	tumor necrosis factor
IL	interleukin
IFN	interferon
EPA	eicosapentaeonic acid
HPA	hypothalamic–pituitary–adrenal
MAP2	microtubule associated protein type-2
rTMS	repetitive transcranial magnetic stimulation
CB	cannabinoid
